# Bicycling crash circumstances vary by route type: a cross-sectional analysis

**DOI:** 10.1186/1471-2458-14-1205

**Published:** 2014-11-22

**Authors:** Kay Teschke, Theresa Frendo, Hui Shen, M Anne Harris, Conor CO Reynolds, Peter A Cripton, Jeff Brubacher, Michael D Cusimano, Steven M Friedman, Garth Hunte, Melody Monro, Lee Vernich, Shelina Babul, Mary Chipman, Meghan Winters

**Affiliations:** School of Population and Public Health, University of British Columbia, 2206 East Mall, Vancouver, BC Canada; School of Occupational and Public Health, Ryerson University, Toronto, ON Canada; Institute for Resources, Environment and Sustainability, University of British Columbia, Vancouver, BC Canada; Department of Mechanical Engineering, ICORD and the Brain Research Centre, University of British Columbia, Vancouver, BC Canada; Department of Emergency Medicine, University of British Columbia, Vancouver, BC Canada; School of Public Health, University of Toronto, Toronto, ON Canada; Emergency Medicine, University Health Network, Toronto, ON Canada; British Columbia Injury Research and Prevention Unit, Vancouver, BC Canada; Faculty of Health Sciences, Simon Fraser University, Burnaby, BC Canada

**Keywords:** Bicycling injuries, Bike lanes, Traffic accidents

## Abstract

**Background:**

Widely varying crash circumstances have been reported for bicycling injuries, likely because of differing bicycling populations and environments. We used data from the Bicyclists’ Injuries and the Cycling Environment Study in Vancouver and Toronto, Canada, to describe the crash circumstances of people injured while cycling for utilitarian and leisure purposes. We examined the association of crash circumstances with route type.

**Methods:**

Adult cyclists injured and treated in a hospital emergency department described their crash circumstances. These were classified into major categories (collision vs. fall, motor vehicle involved vs. not) and subcategories. The distribution of circumstances was tallied for each of 14 route types defined in an earlier analysis. Ratios of observed vs. expected were tallied for each circumstance and route type combination.

**Results:**

Of 690 crashes, 683 could be characterized for this analysis. Most (74%) were collisions. Collisions included those with motor vehicles (34%), streetcar (tram) or train tracks (14%), other surface features (10%), infrastructure (10%), and pedestrians, cyclists, or animals (6%). The remainder of the crashes were falls (26%), many as a result of collision avoidance manoeuvres. Motor vehicles were involved directly or indirectly with 48% of crashes. Crash circumstances were distributed differently by route type, for example, collisions with motor vehicles, including “doorings”, were overrepresented on major streets with parked cars. Collisions involving streetcar tracks were overrepresented on major streets. Collisions involving infrastructure (curbs, posts, bollards, street furniture) were overrepresented on multiuse paths and bike paths.

**Conclusions:**

These data supplement our previous analyses of relative risks by route type by indicating the types of crashes that occur on each route type. This information can guide municipal engineers and planners towards improvements that would make cycling safer.

## Background

There is renewed interest in promoting bicycling around the world – to increase physical activity in the population, promote city vitality, and reduce traffic congestion, air pollution and greenhouse gases [1]. Evidence shows that the safety and motivators of utilitarian and leisure cycling are influenced by route infrastructure [2–10]. Bike-specific facilities that reduce interactions with motor vehicle traffic have lower crash risk for cyclists [2–6]. Such facilities also encourage cycling [7–10]. As this evidence has grown, many cities have begun to build new facilities that offer dedicated space for cyclists [1, 11]. Crashes may occur on any route type, but the circumstances (e.g., falls, collisions) may differ. Understanding these differences will help planners and engineers select and design cycling routes in a way that maximizes safety.

A number of cycling injury studies have reported crash circumstances. Most report whether a crash was a collision with a motor vehicle or not [12–18]. Many report other collisions (e.g., with pedestrians, cyclists, animals, or objects) and falls [12, 14, 16–19]. There is considerable variance in the proportions of various crash circumstances reported from study to study. This may be a result of different cycling infrastructure in the locations studied, but this has rarely been investigated or described [18, 20].

Differences in crash circumstances may also be related to study design, for example the population or mode of cycling being investigated. Bicycling is a term that represents an array of activities that includes not only cycling as a mode of utilitarian or leisure travel where safety is desired and expected, but also as a sport (e.g., road racing, mountain biking, cyclo-cross, BMX, trick riding) where risk-taking is intentional and part of the challenge [21]. Crashes that occur during these very different activities are best examined separately. Unfortunately most administrative data on bicycling injuries offer two extremes: a narrow focus on motor vehicle crashes or a breadth that includes all types of cycling together. Transportation data typically only count collisions with motor vehicles [13, 22]. Hospitalization data usually captures all cyclist crashes, including injuries incurred in deliberately risky cycling sports and in utilitarian or leisure cycling [15, 23]. Studies using primary data collection may also mix these [2, 16].

We previously conducted a study of 690 cyclists injured in two of Canada’s largest cities, Toronto and Vancouver: the Bicyclists’ Injuries and the Cycling Environment Study [3, 4]. Its primary purpose was to examine the relative risks of cycling injury by route type and other infrastructure features. Data were collected from cyclists who were injured seriously enough to be treated in a hospital emergency department. We excluded crashes incurred in mountain biking, racing and trick riding, so the study focused on cycling as a mode of utilitarian and leisure travel using urban transportation infrastructure designed by planners and transport engineers. The relative risk results are outlined in detail elsewhere [3, 4], but in brief, we found that injury risks were highest on major streets with car parking and no bike infrastructure, and were lower on cycle tracks, bike lanes, local streets and bike paths.

To understand how the injuries occurred, here we describe elements of the crash circumstances observed in the study and examine whether the circumstances differed on 14 route types defined in the main study analysis [3].

## Methods

The study methods were reviewed and approved by the human subjects ethics review boards of the University of British Columbia, the University of Toronto, St. Paul’s Hospital, Vancouver General Hospital, St. Michael’s Hospital, and the University Health Network (Toronto General Hospital and Toronto Western Hospital). All participants gave written informed consent before taking part in the study.

Study procedures have been described in detail elsewhere [3, 24]; the following is a summary. The study population consisted of adult (≥19 years) residents of Toronto and Vancouver who were injured while riding a bicycle in the city and treated within 24 hours in the emergency departments of the hospitals listed above between May 18, 2008 and November 30, 2009. All hospitals were located in central business districts, and one in each city was a regional trauma centre.

Eligible participants were interviewed in person by trained interviewers, using a structured questionnaire (http://cyclingincities.spph.ubc.ca/files/2011/10/InterviewFormFinal.pdf) as soon as possible after the injury to maximize recall. Crash circumstances were derived from participants’ answers to the following questions:

- In your own words, please describe the circumstances of the injury incident. (response open-ended)
- Was this a collision between you and a motor vehicle, person, animal or object (including holes in the road)? (response options: yes, no)
- If yes, what did you collide with? (response options: car, SUV, pick-up truck, or van; motorcycle or scooter; large truck; bus or streetcar; pedestrian; cyclist; animal; other non-motorized wheeled transport; pot hole or other hole; streetcar or train track; other (specify))

**Figure 1:**
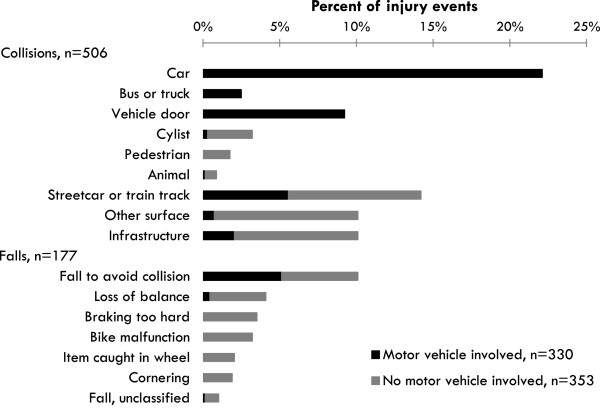
**Crash circumstances, stratified by collisions and falls, and by motor vehicle involvement or not.**

A classification system for the crash circumstances (Figure 1) was developed based on a review of other systems in the injury literature [12–19] and the range of responses to the questions above. Each participant’s answers to the questions were reviewed and classified by two study investigators (TF, KT), blind to route type. Differences in initial classifications were reviewed and adjudicated (KT).

We determined features of the crash site and of a randomly selected control site located along the route of the trip during which the injury occurred. The probability that specific route types would be selected as controls was proportional to their relative lengths on the trips (e.g., on a 4-km trip, there would be a 25% chance of selecting a control site on a 1-km section that was on a bike path). Cumulated over all trips, the control sites provide an estimate of study participants’ exposure to the various route types.

**Figure 2:**
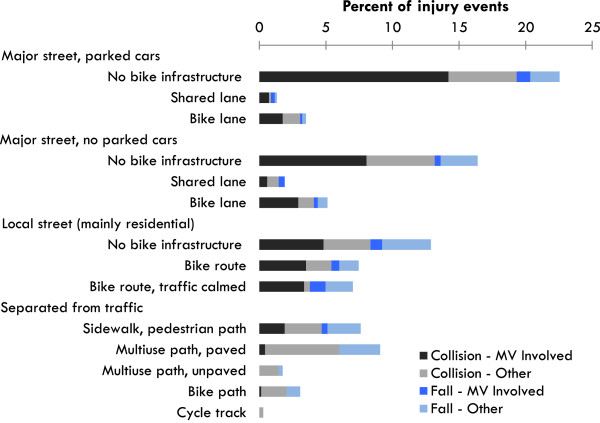
**Route types where the 683 injury events occurred, stratified by broad crash circumstance categories.** MV = motor vehicle.

Data were collected at every injury and control site via structured observations by trained personnel blinded to site status (http://cyclingincities.spph.ubc.ca/files/2011/10/SiteObservationFormFinal.pdf). These observations were used to classify the sites into 14 route types (Figure 2) and provide contextual information such as traffic volumes and speeds [3]. Observations were conducted at a time that conformed as closely as possible to the time of the crash (i.e., season; weekday vs weekend; morning rush, midday, afternoon rush, evening, night).

Data analyses were performed using JMP 10 (SAS Institute, Cary, NC) and R (http://www.r-project.org). We tallied the crash circumstances and cross-tabulated them with route type. We examined associations between crash circumstances and route type by calculating the ratio of observed to expected injury events for each crash circumstance and route type combination. Expected events were calculated two ways: 1) using the distribution of controls sites (reflecting exposure) by route type, and 2) using the distribution of injury sites by route type:

Expected_1_ = all control sites with that route type * all injury events with that crash circumstance/all injury events

Expected_2_ = all injury sites with that route type * all injury events with that crash circumstance/all injury events

Confidence intervals (95%) for the ratio of observed to expected events were calculated using the R function prop.test. Since there were zero injury events for some circumstances and route types, the commonly used normal approximation was not appropriate. Instead, the Wilson score with continuity correction was used to obtain the 95% CI for each proportion [25, 26].

## Results

The study recruited 690 injured cyclists (414 in Vancouver, 276 in Toronto). Most participants were men (59%), younger than 40 years (62%), well-educated (75% with a post-secondary diploma or degree), employed full time (69%), regular cyclists (88% cycled ≥52 times per year). Most of the trips during which the injuries occurred were utilitarian in nature (74%), on weekdays (77%), during daylight hours (78%), and short (68% <5 km) [3].

Seven of the 690 injured cyclists could not recall enough about their crash to classify it for this analysis. Of the available 683 crashes, 506 were classified as collisions and 177 as falls. Figure 1 lists 16 detailed crash circumstance categories, and further stratifies them according to whether a motor vehicle was involved. Motor vehicles were involved directly in 231 (33.8%) collisions, with cars, buses, trucks or vehicle doors. They were also involved indirectly when cyclists took avoidance manoeuvres that resulted in other collisions or falls (99 additional crashes, 14.5%). The top crash circumstances were collisions with cars (22.1% of crashes), streetcar (tram) tracks (14.2%), other surfaces (10.1%), infrastructure (10.1%), vehicle doors (9.2%), and falls to avoid collisions (10.1%). Crashes with other cyclists, pedestrians or animals were rare (total = 5.9%).

Figure 2 and Table 1 list the 14 route types where the 683 injury events occurred. To describe these route types, we measured traffic and speeds. Median motor vehicle traffic and median speeds were higher on major streets than local streets (~900 vs. 50 vehicles/hour and ~40 vs. 30 km/h, respectively). Median bike traffic was highest on cycle tracks (114/h), then bike lanes and multi-use paths (60-78/h), then shared lanes, local street bikeways and bike paths (36-48/h), and lowest on streets with no bike infrastructure (0-24/h).

**Table 1:** **Observed injury events classified by crash circumstance and route type**

	Injury sites	Motor vehicle (excluding door)	Motor vehicle door	Pedestrian, cyclist or animal	Streetcar (tram) or train tracks	Other surface	Infrastructure	Fall to avoid collision	Other fall
	683	168	63	40	97	69	69	69	108
Major street, with parked cars									
No bike infrastructure	155	42	31	2	49	6	3	8	14
^A^Shared lane	9	3	2	-	-	-	1	2	1
Bike lane	24	8	4	1	2	4	2	2	1
Major street, no parked cars									
No bike infrastructure	112	24	12	5	28	9	12	4	18
^A^Shared lane	13	1	2	2	2	2	1	3	-
Bike lane	35	14	1	1	5	2	5	2	5
Local street (mainly residential)									
No bike infrastructure	88	24	5	4	5	13	6	5	26
Bike route	51	18	4	1	1	7	6	5	9
Bike route, with traffic calming	48	19	2	2	-	2	1	12	10
Separated from traffic									
Sidewalk or other pedestrian path	52	12	-	2	2	7	9	9	11
Multiuse paths, paved	61	3	-	12	3	9	13	13	8
Multiuse paths, unpaved	12	-	-	1	-	7	2	1	1
Bike path	21	-	-	6	-	-	8	3	4
^B^Cycle track	2	-	-	1	-	1	-	-	-

- no injury events with this crash circumstance on this route type.

^A^Shared lanes include traffic lanes marked with sharrows or shared HOV lanes.

^B^Cycle tracks run alongside major streets but are physically separated from them, except at intersections. They are also called “separated bike lanes” or “protected bike lanes”.

The dominant route types where crashes occurred were major streets with no bike infrastructure (with or without parked cars, 22.5% and 16.4% respectively), residential streets with no bike infrastructure (12.9%), and off-street multiuse paths (9.1%). Note that the distribution of injury events by route type was influenced both by where people cycled and the risk of a specific route type (relative risks by route type are described in detail in our earlier paper and reported in brief in Table 2 here) [3]. Motor vehicle involvement in collisions and falls featured most prominently on major streets with parked cars, and almost not at all on routes separated from traffic. A minority of all crashes occurred at intersections (31%), though a higher proportion of motor vehicle collisions were at intersections (53%) (data not shown).

**Table 2:** **Ratio of observed to expected injury events for each crash circumstance and route type combination**

	Odds Ratio (relative risk of injury) by route type [3] ^A^		Ratios of observed to expected _1_injury events (and 95% confidence intervals) ^B^							
		Control sites	Motor vehicle (excluding door)	Motor vehicle door	Pedestrian, cyclist or animal	Streetcar (tram) or train track	Other surface	Infrastructure	Fall to avoid collision	Other fall
		683	168	63	40	97	69	69	69	108
Major street, with parked cars										
No bike infrastructure	1.0 reference	114	**1.5** ^B^(**1.1-1.9**)	**3.0** (**2.1-4.0**)	0.3 (0.1-1.2)	**3.0** (**2.4-3.7**)	0.5 (0.2-1.2)	**0.3** (**0.1-0.8**)	0.7 (0.3-1.4)	0.8 (0.5-1.3)
^C^Shared lane	0.78	7	1.7 (0.5-3.2)	3.1 (0.6-7.6)	0 (0–7.5)	0 (0–3.1)	0 (0–4.4)	1.4 (0.1-5.7)	2.8 (0.5-6.9)	0.9 (0.1-3.7)
Bike lane	0.53	27	1.2 (0.6-2.1)	1.6 (0.5-3.8)	0.6 (0–3.6)	0.5 (0.1-1.8)	1.5 (0.5-3.4)	0.7 (0.1-2.6)	0.7 (0.1-2.6)	0.2 (0–1.3)
Major street, no parked cars										
No bike infrastructure	*0.65	116	0.8 (0.6-1.2)	1.1 (0.6-1.9)	0.7 (0.3-1.8)	**1.7** (**1.2-2.3**)	0.8 (0.4-1.5)	1.0 (0.6-1.8)	0.3 (0.1-0.9)	1.0 (0.6-1.5)
^C^Shared lane	0.66	12	0.3 (0–1.6)	1.8 (0.3-5.3)	2.9 (0.5-8.4)	1.2 (0.2-3.5)	1.7 (0.3-4.9)	0.8 (0–4.0)	2.5 (0.7-5.7)	0 (0–1.9)
Bike lane	*0.47	46	1.2 (0.7-1.9)	0.2 (0–1.4)	0.4 (0–2.2)	0.8 (0.3-1.7)	0.4 (0.1-1.6)	1.1 (0.4-2.4)	0.4 (0.1-1.6)	0.7 (0.3-1.5)
Local street (mainly residential)										
No bike infrastructure	*0.44	115	0.9 (0.6-1.2)	0.5 (0.2-1.1)	0.6 (0.2-1.6)	**0.3** (**0.1-0.7**)	1.1 (0.6-1.9)	0.5 (0.2-1.1)	0.4 (0.2-1.0)	1.4 (0.9-2.0)
Bike route	*0.53	56	1.3 (0.8-1.9)	0.8 (0.3-2.0)	0.3 (0–1.9)	**0.1** (**0**–**0.8**)	1.2 (0.6-2.4)	1.1 (0.4-2.2)	0.9 (0.3-2.0)	1.0 (0.5-1.8)
Bike route, with traffic calming	0.59	46	**1.7** (**1.1-2.3**)	0.5 (0.1-1.7)	0.7 (0.1-2.7)	**0** (**0**–**0.7**)	0.4 (0.1-1.6)	0.2 (0–1.3)	**2.6** (**1.5-4.1**)	1.4 (0.7-2.3)
Separated from traffic										
Sidewalk, pedestrian path	0.73	47	1.0 (0.6-1.7)	0 (0–1.0)	0.7 (0.1-2.7)	0.3 (0.1-1.1)	1.5 (0.7-2.9)	1.9 (1.0-3.3)	1.9 (1.0-3.3)	1.5 (0.8-2.4)
Multiuse paths, paved	0.75	55	**0.2** (**0.1-0.7**)	**0** (**0**–**0.9**)	**3.7** (**2.1-6.0**)	0.4 (0.1-1.1)	1.6 (0.8-2.9)	**2.3** (**1.4-3.7**)	**2.3** (**1.4-3.7**)	0.9 (0.4-1.7)
Multiuse paths, unpaved	0.63	11	0 (0–1.3)	0 (0–3.5)	1.6 (0.1-7.3)	0 (0–2.3)	**6.3** (**3.1-8.7**)	1.8 (0.3-5.2)	0.9 (0.1-4.2)	0.6 (0–2.7)
Bike path	0.54	21	**0** (**0**–**0.8**)	0 (0–2.1)	**4.9** (**2.1-8.9**)	0 (0–1.4)	0 (0–1.9)	**3.8** (**1.9-6.1**)	1.4 (0.4-3.7)	1.2 (0.4-2.7)
^D^Cycle track	*0.12	10	0 (0–1.4)	0 (0–3.7)	1.7 (0.1-7.8)	0 (0–2.4)	1.0 (0.1-4.5)	0 (0–3.4)	0 (0–3.4)	0 (0–2.2)

^A^Odds ratios (relative risks of injury) by route type are from a previous analysis [3] and are provided for reference only. Asterisks indicate risk of injury for this route type was significantly lower than on major streets with parked cars and no bike infrastructure (the reference category).

^B^Ratios of observed to expected_1_ injury events and confidence intervals in bold when statistically significantly different from 1.0. Expected_1_ based on exposure to route type, estimated via randomly selected control sites on the trip route.

^C^Shared lanes include traffic lanes marked with sharrows or shared HOV lanes.

^D^Cycle tracks run alongside major streets but are physically separated from them, except at intersections. They are also called “separated bike lanes” or “protected bike lanes”.

Statistical significance, p ≤ 0.05.

Table 1 shows a cross-tabulation of crash circumstances by route type. To ensure numbers for subsequent analyses, some circumstances shown in Figure 1 were grouped into larger categories (circumstances with <5% of crashes). There were no collisions involving motor vehicle doors on any of the route types separated from traffic. There were no collisions with motor vehicles or with streetcar or train tracks on unpaved multiuse paths, bike paths, or cycle tracks.

Table 2 reports associations between crash circumstance and route type via the ratio of observed to expected injury events, using the distribution of controls sites (reflecting exposure) by route type (Expected_1_). All crash circumstances except “other fall” were associated with route type. Collisions involving motor vehicles, including motor vehicle doors, were consistently higher than expected for all major street route types with parked cars, significantly so where there was no infrastructure for bikes. This excess was not observed on major streets without parked cars. Streetcar and train track collisions were significantly higher than expected on major streets without bike infrastructure, whether or not there were parked cars. Local street bike routes with traffic calming had significantly more motor vehicle collisions and falls to avoid collisions than expected. Paved multi-use paths and bike paths had more collisions than expected involving infrastructure and pedestrians, cyclists or animals. Paved multi-use paths had more falls to avoid collisions than expected. Unpaved multi-use paths had more collisions involving surfaces than expected.

We also calculated observed to expected injury events using the distribution of injury sites by route type (Expected_2_, data not shown). Using this method, associations between crash circumstance and route type did not differ substantively from those described above.

## Discussion

In this study, we examined a large number of crash circumstances and considered their distributions across 14 route types. Of the 683 crashes characterized, 34% were direct collisions with motor vehicles, 6% were collisions with pedestrians, cyclists, or animals, 34% were collisions with infrastructure or surface features, and 26% were falls. Crash circumstances were distributed differently by route type, for example, motor vehicle and tram track collisions were overrepresented on major streets, and infrastructure or other surface collisions were overrepresented on off-street routes. Below, our results for each circumstance type is considered in light of other research.

### Crashes involving motor vehicles

Understanding collisions with motor vehicles is particularly important because they typically result in more severe injuries [2, 15, 27] and concern about collisions with motor vehicles deters cycling [8, 9]. In this study, 34% of the injury events were direct crashes with motor vehicles. Studies of hospital visits in comparable jurisdictions with little specialized bicycling infrastructure have found similar proportions: 27% in the US [15]; 31% in France [12]; and 34% in New Zealand [17]. Others have reported lower proportions of collisions with motor vehicles: 9% in Sweden [14]; 14% in Australia [16]; 18% in the Netherlands [19]; and 21% in South Korea [18]. These lower proportions may result from different case definitions (inclusion of less serious injuries and sports cycling injuries, as in the Australian study) [16] or the bicycling facilities available in the area (routes that separate cyclists from motor vehicles, as in Sweden, the Netherlands and Korea) [14, 18, 19].

The potential for cycling infrastructure to reduce crashes between cyclists and motor vehicles is observed in our results. Collisions with motor vehicles represented 40% of all crashes on streets. Major streets with parked cars had more crashes with vehicles than expected, including those with vehicle doors. In contrast, collisions with motor vehicles on routes separated from traffic were rare (10%). There has been concern that cycle tracks and other separated infrastructure might pose a special risk to cyclists when they eventually meet traffic at intersections [5]. Our results show that even if that were the case, the overall benefit of separation is maintained. Other studies found similar benefits to separated infrastructure. A study in South Korea [18] found that 40% of bike crashes on regular roadways were with motor vehicles, compared to only 4.4% of those on bike lanes (typically separated). A study in Australia found that 35% of bike crashes in traffic involved motor vehicles, compared to only 11% of those on other facilities (bike lanes, shared paths, footpaths) [20].

A number of studies have tallied collisions with opening doors of parked vehicles (“doorings”). In a Swedish study, “doorings” accounted for 4.3% of collisions with motor vehicles [22], in a Dutch study, 3% of single party crashes [19] and in Australian studies, 2.2% of surveyed cyclists, 3.1% of hospital presentations, and 8.1% of police reported crashes [16, 28]. These proportions are all considerably lower than we found (10% of all crashes, 27% of motor vehicle collisions). The Australian study included mountain biking and racing injuries, likely influencing the low proportion there [16]. In Sweden and the Netherlands, the prevalence of well designed, usually separated facilities on major streets likely made collisions with vehicle doors rare.[19, 22] In Vancouver and Toronto at the time of our study, cycling between parked and moving cars was often the only option on major roads, even where there were painted bike lanes or shared lanes.

Tallying direct collisions with motor vehicles may not provide a complete picture of motor vehicles’ influence on cycling injuries. In the Australian survey, cyclists reported that 5% of crashes involved motor vehicle collision avoidance [16]. In our study, 15% of cases involved crashes to avoid a motor vehicle, so in total, motor vehicle interactions were responsible for half the crashes. Separated routes prevent these interactions (except at intersections) and can prevent whole classes of crashes such as doorings [3, 5].

### Crashes involving people or animals

A common concern with separated and off-street bike facilities is collisions with other cyclists, pedestrians, or animals. Only 5.9% of the injury events in this study involved such collisions. Similar low proportions were identified in France and New Zealand [12, 17], but in South Korea where cycle lanes were more common, 15% of crashes were with other cyclists and 3% with pedestrians [18]. An Australian survey also reported a higher proportion of crashes between cyclists (11%), though one-quarter of their survey cohort were racing cyclists who may collide during training and races [16].

We found more crashes involving people or animals than expected on multi-use paths. Multi-use paths are designated for both pedestrians and cyclists, so this result is not a surprise. Multi-use paths also had more falls to avoid collisions than expected, most to avoid other cyclists or pedestrians. Another study reported higher proportions of cyclist and pedestrian collisions or collision-avoidance crashes on multi-use paths [20].

Bike only paths also had more collisions than expected with cyclists and pedestrians (in equal numbers), suggesting that the delineation of the path for cyclists may not have been clear or that heavy pedestrian traffic overflowed to the cyclist side. Bike paths did not have a problem with falls to avoid collisions, suggesting they did function better than multi-use paths.

### Crashes with infrastructure and surface features

Much more common than collisions with people or animals were those with infrastructure or surface features. These contributed 34% of injury events, the same as motor vehicle collisions. This group comprised many crash circumstances, most related to route type, and likely preventable via design solutions.

Crashes on streetcar (tram) or train tracks made up 14% of all events, and were in excess on major streets. Toronto has an extensive streetcar system in its central business district, not separated from traffic along most streets. In our previous analyses, we found greatly increased relative risk where streetcar tracks were present [3, 4]. Streetcar track crashes involved wheels being caught in the slot or slipping on the rail surface. Two recent reports from Europe noted the issue of tram tracks [19, 29]. Physically separated bike lanes or streetcar lanes are potential design changes that would greatly reduce this type of crash. Crossings would still be needed at intersections, but in our study two-thirds of the crashes involving tracks were not at intersections.

While streetcar or train tracks were a problem on major city streets, other surfaces (10% of crash circumstances) were involved in crashes across all route types, with unpaved multi-use paths showing a strong excess. Crashes with surfaces involved bumps, potholes, gravel, icy or wet surfaces, and vegetation such as roots or leaves, pointing to the importance of route maintenance. Some studies tallied surface feature crash circumstances: 18% in Australia [16]; 23% (including tram rails) in the Netherlands [19]; and 21% (including tracks) in Belgium [29]. These proportions are similar to the total of streetcar track and other surface crashes we found (24%).

Infrastructure such as curbs, concrete barriers, walls, fences, railings, furniture, boulders, speed bumps, and stairs contributed 10% of crash circumstances, and were overrepresented particularly on paved multi-use paths and bike paths. In our previous analyses of relative risks by route type, we found that multi-use and bike paths were not as safe as cycle tracks and local street bikeways with traffic diversion [4]. A reason may be that such paths were often designed to be interesting (e.g., with street furniture and curves) and to direct traffic (using bollards, signage, curbs and fences to prevent motor vehicle ingress or to separate pedestrians and cyclists). In measurements taken at injury and control sites, 5 to 10% of bike and multi-use paths had poor forward visibility, but this was not a problem on on-street routes. The crashes with infrastructure suggest a rethink of multi-use and bike path design to provide straight, wide and obstacle-free passage for cyclists. In other studies, infrastructure was involved in 8 to 31% of crashes [12, 16, 18, 19]. A South Korean study tallied crashes with obstacles by route type; it found similar proportions (~10%) on both bike lanes and roads [18].

### Falls

Falls to avoid collisions contributed 10% of crash circumstances. About half (N = 34) were to avoid motor vehicles, 16 to avoid pedestrians, 8 to avoid other cyclists, 10 to avoid infrastructure or surface features, and 1 to avoid an animal. Excesses were observed on shared facilities (shared lanes on streets, multi-use paths) and sidewalks, reinforcing the importance of bike-specific infrastructure [2–4].

Collision avoidance falls were also in excess on local street bike routes with traffic calming, most to avoid motor vehicles. Two types of traffic calming were observed in our study: traffic diversion (full or partial barriers to motor vehicles at intersections with arterials) and traffic slowing (speed humps, traffic circles) [4]. Traffic circles are small diameter (6–8 m) roundabouts used at local street intersections. They had higher relative risk of injury in our earlier analyses [4], in part because drivers did not observe cyclists or did not know who had the right of way. Traffic circles also presented a difficult-to-negotiate obstacle to cyclists. In contrast, bike routes with traffic diversion had very low relative risk of injury in our earlier analyses [4], suggesting this is a better traffic calming method. A British study found a benefit to cyclists of traffic slowing; techniques used (speed humps, chicanes, raised junctions) only partly overlapped with those observed in our study, reinforcing the importance of understanding the effects of specific elements [30]. Raised junctions have been shown to greatly reduce cycling injuries at intersections [19], but these were not observed in our study.

Our category “other falls” (16% of crash circumstances) included loss of balance, braking too hard, bike malfunctions, having an item caught in the wheel and cornering. This crash category was the only one not related to route type. This is reasonable, since these falls represented either problems with the bicycle itself or with bicycling operations.

### Single party (bicyclist only) crashes

Some studies classify crashes as multi-party vs. single party (bicyclist only) crashes. Single party is interpreted as any crash not involving a direct collision with a motor vehicle, pedestrian, cyclist or animal. By this standard, 60% of the crashes in our study were single party crashes. Schepers [19] reviewed data from several countries and reported that 60 to 90% of crashes involving hospital treatment were single cyclist crashes. Our study is at the low end of these results, likely reflecting both the case definition (urban cycling) and the types of routes available to cyclists in Toronto and Vancouver (typically on street mixed with motor vehicle traffic). The above definition of single party omits collision avoidance crashes that do not result in direct collisions with other parties. If we include collision avoidance crashes as multi-party crashes, only 42% remain single party in our study. An Australian study [20] also found that single party crashes were considerably lower once collision avoidance was taken into account (52%).

### Strengths and limitations

This study adds to the small base of evidence examining the distribution of crash circumstances in an urban cycling context [12, 18, 20]. It is the first to report observed to expected crash circumstances by route type (controlling for exposure). It examined 14 route types, many more than previous studies, though this meant that some route types had small numbers of injury events, so that confidence intervals were wide for observed to expected ratios.

We included injuries serious enough to require a hospital visit: treatment in an emergency department or hospital admission, but the most serious injuries (including deaths) were not included because routes and circumstances could not be reported. Hospital-based case identification allowed a broad array of crash circumstances to be captured beyond motor vehicle collisions. Others have reported injuries with hospital identification, providing a basis for comparison [12–15, 17–19]. We restricted cases to those injured while cycling for utilitarian or leisure travel by excluding cases injured during risk-taking sports like mountain biking and racing. This restriction provided a clear delineation of the focus: on cycling for which urban transportation engineers design route infrastructure. Other studies did not have such restrictions and sports injuries may have been substantial, particularly in countries such as the United States, Australia and New Zealand [13, 15, 16, 23].

We classified crash circumstances using classes similar to those in other studies, although each study had variations [12–19]. Collisions with motor vehicles or not is the most frequent basis for classification. We tallied crashes with vehicle doors as a separate category and also tallied motor vehicle involvement in crashes that did not end in a direct collision with a vehicle. Another common basis for classification is collision vs. fall. In collisions, we included crashes with surface features because most of these crashes involved a dramatic change in motion after striking the feature. Some might consider these falls; our separate tally of streetcar track and other surface crashes allows others to do their own calculations. There are other methods of classifying crashes, for example, based on travel movements or collision partner responsibility, but our data did not allow these [31].

Crash circumstances in this study were based on a description of the event by the injured cyclist. This is true of most studies classifying crashes, including surveys of cyclists and studies using hospital coding of injury events [12, 14–18]. The results therefore rely on the accuracy of participants’ recall. To minimize problems related to recall, we excluded cyclists who could not remember their injury event, we interviewed subjects as soon as possible after the crash (50% completed within 4.9 weeks, 75% within 7.7), and we did not ask for comments about fault. Some injury data, particularly from police or transportation agencies, may include reporting by all parties in the crash, witnesses, and investigators [13, 22].

## Conclusions

In the Bicyclists’ Injuries and the Cycling Environment study in Toronto and Vancouver, about one-third of crashes were collisions with motor vehicles (including “doorings”), one-third collisions with infrastructure and surface features, and a small proportion collisions with cyclists, pedestrians and animals. All collision circumstances, and falls to avoid collisions, were related to route type. Our results reinforce the importance of providing bicycle-specific facilities such as cycle tracks alongside major streets and bike paths off-street. They demonstrate the value of not placing cyclists between parked and moving vehicles on major streets to reduce the chance of being hit by a door. They show the value of separation from streetcar (tram) tracks, via cycle tracks or separated streetcar lanes. They shed light on problems with off-street bike paths and multi-use paths, where collisions with infrastructure and surface features were elevated. Such facilities are very attractive to people of all ages and abilities; removing obstacles, providing clear sight lines and ensuring routine maintenance should improve their safety.

Many cities are trying to encourage cycling, and safety is a key motivator [7, 9]. Understanding crash circumstances on the various routes types will help transportation planners and engineers target improvements to make cycling safer.
